# Differences in the Profile of Circulating Immune Cell Subsets in Males with Type 2 Cardiorenal Syndrome versus CKD Patients without Established Cardiovascular Disease

**DOI:** 10.3390/biomedicines11041029

**Published:** 2023-03-27

**Authors:** Anila Duni, Athanasios Kitsos, Aris Bechlioulis, Georgios S. Markopoulos, Lampros Lakkas, Gerasimos Baxevanos, Michail Mitsis, George Vartholomatos, Katerina K. Naka, Evangelia Dounousi

**Affiliations:** 1 Department of Nephrology, School of Health Sciences, Faculty of Medicine, University of Ioannina and University Hospital of Ioannina, GR 45500 Ioannina, Greece; anikristduni@yahoo.com (A.D.); thkitsos@hotmail.com (A.K.); 2 Kidney Transplant Unit, Department of Surgery, Faculty of Medicine, School of Health Sciences, University of Ioannina and University Hospital of Ioannina, GR 45500 Ioannina, Greece; mmitsis@uoi.gr; 3 Second Department of Cardiology, Faculty of Medicine, School of Health Sciences, University of Ioannina and University Hospital of Ioannina, GR 45500 Ioannina, Greece; md02798@yahoo.gr (A.B.); ftpcavalier52@gmail.com (L.L.); drkknaka@gmail.com (K.K.N.); 4 Laboratory of Haematology—Unit of Molecular Biology and Translational Flow Cytometry, University Hospital of Ioannina, GR 45500 Ioannina, Greece; geomarkop@gmail.com (G.S.M.); gbaxevanos@gmail.com (G.B.); gvarthol@gmail.com (G.V.)

**Keywords:** cardiorenal syndrome, immune cell subsets, proinflammatory CD14++CD16+ monocytes, CD4+ T-cells, CD8+ T-cells, T regulatory cells, natural killer cells

## Abstract

Maladaptive activation of the immune system plays a key role in the pathogenesis of chronic kidney disease (CKD). Our aim was to investigate differences in circulating immune cells between type 2 cardiorenal syndrome (CRS-2) patients and CKD patients without cardiovascular disease (CVD). CRS-2 patients were prospectively followed up, with the primary endpoint being all-cause and cardiovascular mortality. Method: A total of 39 stable males with CRS-2 and 24 male CKD patients matched for eGFR (CKD-EPI) were enrolled. A selected panel of immune cell subsets was measured by flow cytometry. Results: Compared to CKD patients, CRS-2 patients displayed higher levels of proinflammatory CD14++CD16+ monocytes (*p* = 0.04) and T regulatory cells (Tregs) (*p* = 0.03), lower lymphocytes (*p* = 0.04), and lower natural killer cells (*p* = 0.001). Decreased lymphocytes, T-lymphocytes, CD4+ T-cells, CD8+ T-cells, Tregs, and increased CD14++CD16+ monocytes were associated with mortality at a median follow-up of 30 months (*p* < 0.05 for all). In a multivariate model including all six immune cell subsets, only CD4+ T-lymphocytes remained independent predictors of mortality (OR 0.66; 95% CI 0.50–0.87; *p* = 0.004). Conclusion: Patients with CRS-2 exhibit alterations in immune cell profile compared to CKD patients of similar kidney function but without CVD. In the CRS-2 cohort, CD4+ T-lymphocytes independently predicted fatal cardiovascular events.

## 1. Introduction

Maladaptive activation of the immune system plays an essential role in the pathogenesis of chronic kidney disease (CKD) and cardiovascular disease (CVD). A significant body of data from animal and human research indicates that inflammation and immunological pathways are implicated in all aspects of CVD phenotypes and have been well-established in atherogenesis, viral myocarditis, and inflammatory cardiomyopathy [1]. 

The hallmark of CKD is represented by the classical trial of endothelial dysfunction, oxidative stress, and chronic inflammation, which participate in the pathogenesis and progression of kidney dysfunction [2,3]. The chronic inflammatory state of CKD is mediated and perpetuated by an intricate interaction of multiple immune mediators as well as cellular components of the innate and adaptive immune systems [2,3]. Chronic kidney disease progression itself is associated with complex alterations in innate immunity and disruption of immune regulatory processes [4,5]. Furthermore, CVD and CKD are closely linked to each other, considering that CKD patients display a significant burden of cardiovascular morbidity and mortality as kidney function progressively exacerbates [6]. Inflammatory and immune pathways appear to participate in the pathogenesis of the entire spectrum of CVD in CKD, including accelerated atherosclerosis, left ventricular hypertrophy, and heart failure [7]. 

Regarding the cellular components of the immune system, specific monocyte subpopulations are an integral part of the inflammatory cascade in both heart failure and CKD [7]. The phenotypically distinct monocyte subsets possessing different functional properties are defined by the expression of CD14 and CD16 antigens on their surface. Accordingly, three monocyte subpopulations are recognized, including the classical CD14++CD16–, the intermediate CD14++CD16+, and the nonclassical CD14+CD16++ monocytes [8]. In CKD patients, the proinflammatory intermediate CD14++CD16+ monocytes exhibit marked uptake of oxidized LDL with low cholesterol efflux capacity, high production of Interleukin (IL)-6, and IL-1β and tumor necrosis factor alpha (TNF-α), and appear to be linked to adverse cardiovascular outcomes [9,10]. Furthermore, evidence suggests that activated monocytes in patients with advanced kidney dysfunction are involved in the induction of the intracardiac renin-angiotensin system and tissue expression of fibroblast growth factor (FGF) 23, thus promoting myocardial remodeling [10]. 

The lymphocyte subpopulations are currently receiving increasing attention for their role in heart failure and CKD. Thus, recent evidence indicates that CD4+ T cells promote the transition from hypertrophy to heart failure during chronic pressure overload, whereas CKD is associated with the accumulation of proinflammatory T-lymphocytes that contribute to myocardial dysfunction [11,12]. On the other hand, regulatory T-lymphocytes (Tregs), which are active players in the maintenance of immune homeostasis and tolerance, display reduced numbers and impaired function in CKD and in a variety of cardiovascular diseases, including atherosclerosis, hypertension, and left ventricular remodeling following myocardial infarction [13,14]. B-lymphocytes are diffusely depleted in uremia, and the uremic milieu induces B-cell resistance to B-cell differentiation and survival factors such as IL-7 and BAFF [15]. According to recent data, B-lymphocyte populations might be associated with an augmented burden of atherosclerosis and left ventricular remodeling in elderly patients in advanced stages of CKD [16,17]. Natural killer (NK) cells represent a bridge between the innate and acquired arms of the immune system. NK cells are abundant in the necrotic cores of atherosclerotic plaques; however, their pathophysiological role has not yet been clarified [18]. Although studies on NK cells in heart failure and CKD are limited, available data indicate that reduced circulating NK cells with impaired activity are found in patients with heart failure and end-stage kidney disease [19,20]. During the last two decades, the term cardiorenal syndrome (CRS) has been introduced and established to mark the tight interaction between the heart and the kidneys [21]. Thus, the acute or chronic dysfunction of the heart or kidneys might lead to acute or chronic impairment in the function of the other organ [21]. Accordingly, five clinical subtypes of CRS have been defined with type 1 CRS (CRS-1) and type 2 CRS (CRS-2) referring to acute and chronic heart failure causing acute kidney injury (AKI) and CKD, respectively, whereas type 3 CRS (CRS-3) and type 4 CRS (CRS-4) refer to AKI and CKD leading to acute and chronic heart failure, respectively [21,22]. Finally, type 5 CRS (CRS-5) represents simultaneous heart and kidney dysfunction in the setting of systemic disease [21,22]. The pathophysiology of CRS, in general, involves complex intertwining pathways, including hemodynamic changes, neurohumoral activation, oxidative stress amplification, and chronic inflammation [23]. The neurohormonal imbalance, together with volume overload, venous congestion, and endothelial dysfunction, further trigger chronic inflammation and immune dysregulation in CRS [24]. Immune system components, including cytokines, toll-like receptors, and innate and adaptive immune system cells, can act as mediators of organ cross-talk and may be involved in the reciprocal dysfunction that occurs in CRS [24]. Available data until now mainly come from experimental and in vitro models and regard immune responses in the acute setting, such as acute myocardial ischemic injury and renal ischemia reperfusion injury [23,25]. In general, the activation of inflammatory pathways in the setting of acute heart failure and AKI promotes the introduction of humoral and cellular mediators in the circulation with adverse systemic clinical implications. Thus, according to present evidence, rapid worsening of cardiac function as occurs in CRS-1 leads to AKI mainly via an interplay of proinflammatory mechanisms with neurohormonal activation, whereas in CRS-2, chronic abnormalities in cardiac function appear to cause progressive CKD by inducing systemic hypoperfusion and kidney congestion [21,24].

Notably, available data until now provide insight mainly into the cardiovascular complications of AKI and especially CKD, that is, CRS-3 and CRS-4. On the other hand, little evidence has been generated until now regarding potential alterations of the cellular components of the immune system in patients with CKD due to heart failure, as occurs in CRS-2. In this pilot study, we focused on the alterations of specific immune cell subsets in the setting of CRS-2. Accordingly, the aim of our study was to investigate the expression of a selected panel of immune cell subsets in the peripheral blood of a cohort of CRS-2 patients and subsequently make comparisons to a group of patients with CKD but without established CVD matched for gender, and estimated glomerular filtration rate (eGFR). In addition, we examined the clinical correlations as well as the prognostic value of specific immune cells with respect to overall and cardiovascular mortality in patients with type CRS-2.

## 2. Materials and Methods

### 2.1. Study Cohort

**Figure 1:**
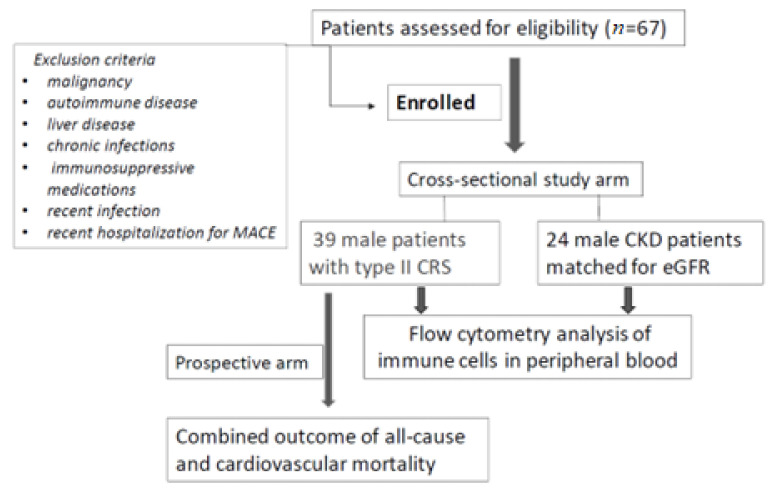
Flowchart of the study.

We conducted an observational, prospective cohort study on 39 stable male patients with CRS-2 under regular follow-up by the outpatient chronic heart failure and CKD clinic of our hospital. Type 2 CRS was defined as chronic abnormalities in heart function leading to kidney injury and/or dysfunction according to the classification of CRS as proposed in the consensus conference on CRS held in Venice, Italy, in September 2008 under the auspices of the Acute Dialysis Quality Initiative (ADQI) [22]. In addition, 24 male patients with CKD but without established CVD and matched for eGFR (CKD-EPI) were enrolled in this study. Exclusion criteria included a history of malignancy, autoimmune disease, liver disease, chronic infections, and current treatment with steroids and other immunosuppressive medications. Additionally, patients with a recent infection and/or recent hospitalization (<1 month) for any major adverse cardiovascular event were excluded from the study (Figure 1). The study protocol was approved by the Ethical Committee of the University Hospital of Ioannina (5/26-3-2020), and fully informed consent was obtained from all participants.

### 2.2. Evaluation of Specific Immune Cell Subsets

**Figure 2:**
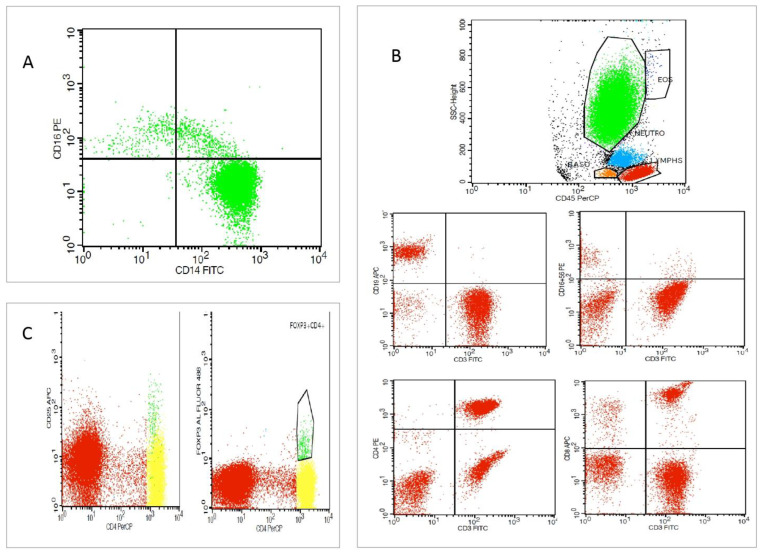
Flow cytometric analysis of a type 2 CRS patient. (**A**) Representative dot plots depicting monocyte subsets (green clolour) with regard to surface expression of CD14 and CD16 in CD14++CD16−, CD14++C16+, and CD14+CD16+ subpopulations. (**B**) Representative dot plots depicting lymphocyte gating (red colour) with B-lymphocytes, T-lymphocytes, and natural killer (NK) cells defined as CD16+CD56+ cells, CD4+ T cells, and CD8+ T cells. (**C**). Representative dot plots depicting T regulatory cells (Tregs) defined as CD4+ FoxP3+ CD25^high^ positive cells (green colour).

The peripheral blood immune cell subsets analysis was performed by flow cytometry (FC) in a 100 μL whole-blood assay within 8 h from sample withdrawal. Ethylenediaminetetraacetic acid (EDTA) tubes were used for the collection of 2 mL of whole blood from patients under standardized conditions. The following conjugated monoclonal antibodies were used for analysis: CD45(BD), CD14(BD), CD16(BD), CD4(BD), CD8(BD), CD56(BD), CD3(BD), CD19(BD), CD25(BD), and Fox-P3 (all from eBioscience, Thermo Fisher Scientific Inc., Waltham, MA, USA). Immune cell subtypes were analyzed using standard techniques with FC (FACSCalibur), Cell Quest, and the FACSDiva software (BD Biosciences, San Jose, CA, USA). An amount of 100 μL of whole blood was placed in FC tubes and incubated with respective antibodies according to the manufacturer’s instructions. An amount of 500 μL of Versalyse ( Beckman Coulter, Brea, CA, USA) was added and incubated for 10 min at room temperature (18–25 °C), protected from light, to lyse red blood cells. Samples were processed immediately for analysis. The data were analyzed using the CellQuest v.3.1 software ( BD Biosciences, San Jose, CA, USA). Accordingly, CD14++CD16−, CD14++CD16+, and CD16+ percentage and the absolute number of cells out of the total monocytes, as well as NK cells (CD3+CD16+56+), CD3- CD19+ B-lymphocytes, CD3+ CD4+ T cells, CD3+CD8+ T cells, and Treg (CD4+CD25+ FoxP3+) absolute values, and percentage out of the total lymphocytes were measured (Figure 2). 

### 2.3. Clinical and Laboratory Assessment

Anthropometric and clinical data were recorded at baseline by patients’ medical records, including comorbidities such as the presence of diabetes mellitus (DM) and medications. Specifically, with regard to patients with type CRS-2, the presence of coronary artery disease (CAD), peripheral artery disease (PAD), and atrial fibrillation was recorded. In addition, common biochemical parameters were measured at baseline in accordance with standard methods applied in the hospital laboratory. Complete blood counts and classical inflammatory markers, including C-reactive protein (CRP) and erythrocyte sedimentation rate (ESR), serum levels of glucose, uric acid, total protein, albumin, total cholesterol, triglyceride, high-density lipoprotein (HDL) cholesterol, low-density lipoprotein (LDL) cholesterol, calcium, phosphorus, intact parathyroid hormone (iPTH), and ferritin were determined. Urinary protein to creatinine ratio (UPCR) assessment was performed in morning spot urine samples. Brain natriuretic peptide (BNP) levels and high sensitivity troponin I (hsTnI) as respective markers of heart failure severity and subclinical myocardial damage were measured in patients with CRS-2. Echocardiographic data from ultrasounds performed by a skilled operator within 1 month from immune cell subset analysis were recorded, including parameters for estimating ventricular function and morphology and for cardiac chamber quantification.

### 2.4. Prospective Follow-Up and Study Endpoint

After baseline evaluation, patients were followed until the end of the established observation period, or the study endpoint was reached, which was defined as a combined outcome of all-cause mortality and cardiovascular mortality.

### 2.5. Statistical Analysis

Descriptive statistics are reported as mean ± standard deviation in normally distributed continuous variables, median and interquartile range in skewed continuous variables, and binary data as frequency percentage. The normal distribution of all continuous variables was tested with the parametric Shapiro–Wilk normality test. Differences between groups were determined by independent samples t-test or nonparametric Mann–Whitney test, in normally and skewed continuous variables, respectively, and the chi-square followed by a Fisher’s exact test for categorical variables (frequency distributions). Correlation analyses were made by assessing the Pearson (R) or the Spearman (Rho) coefficients, as indicated. Linear regression model analysis was used to adjust for confounders (including age) in cell subtype differences among various patient subgroups. Logistic regression analysis was used to identify predictors of mortality among various cell subtypes in CRS patients. Accordingly, CRS patients were categorized as ≥ median value or < median value based on cell subtypes with significant associations in logistic regression analysis. Kaplan–Meier curves were then generated and compared by a log-rank test for each of the immune cell subsets of interest. All endpoint analyses were conducted on a time-to-first-event basis. The SPSS v.23.0 software was applied to analyze all data, and the significance level was set at 0.05 in all cases.

## 3. Results

Table 1 summarizes the main baseline characteristics of the study cohort.

The mean age of patients with CRS-2 was 72 ± 10 years, whereas patients with CKD had a mean age of 66 ± 10 years, respectively (*p* = 0.01). As for the presence of diabetes mellitus, no significant differences were detected between the two patient groups. With regard to indices of renal function, the mean eGFR of patients with CRS-2 and CKD patients was 37 ± 14 and 33 ± 16 mL/min/1.73 m^2^, respectively (*p* = 0.28), whereas the median UPCR was 0.19 g protein/g creatinine (IQR, 0.10–0.52) versus 1.03 (IQR, 0.17–2.09) g protein/g creatinine (*p* = 0.02), respectively. CRS-2 patients displayed lower levels of total cholesterol (147 ± 40 mg/dl vs. 184 ± 41 mg/dL, *p* = 0.001), LDL cholesterol (84 ± 35 vs. 110 ± 44, *p* = 0.01), and triglycerides as compared to patients with CKD. In contrast, no significant differences were found regarding the use of statins. In specific, within the CRS-2 patient group, 29 patients (74.3%) had ischemic cardiomyopathy in the setting of CAD, whereas 5 patients had dilated cardiomyopathy (12.8%). In addition, 13 patients (33%) with CRS-2 had PAD, and atrial fibrillation was present in 26 patients (66%). Left ventricular ejection fraction (EF) was less than 30% in 17 patients (44.7%), whereas with regard to NYHA class, 8 patients (21%) had NYHA class I, 14 patients (38%) had NYHA class II, and 16 patients (41%) had NYHA class III heart failure, respectively. 

### 3.1. Differences in the Profile of Immune Cell Subset Expression between Patients with Type 2 CRS versus CKD Patients

The differences between the peripheral blood levels of immune cell subpopulations between patients with CRS-2 and CKD patients are depicted in Table 2. CRS-2 patients displayed increased levels of proinflammatory intermediate CD14++CD16+ monocytes [41 (IQR, 24–78)/µL] compared to their CKD counterparts [35 (IQR, 18–43)/µL] (*p* = 0.04). A higher Treg percentage was found in CRS-2 patients [2.7% (IQR, 2.0–3.9%)] compared to CKD patients [2.0% (IQR, 1.6–2.6%)] (*p* = 0.03). Lower mean levels of lymphocytes were observed in CRS-2 patients (1557 ± 691/µL) compared to the CKD cohort (1920 ± 545/µL) (*p* = 0.04). Finally, CRS-2 patients displayed lower NK cell counts [148 (IQR, 103–258)/µL] compared to CKD patients [324 (IQR, 179–368)/µL] (*p* = 0.001). Subsequently, we sought to determine whether the differences regarding the expression of immune cells between the two groups remained significant after adjusting for other significant correlates of immune cells in the whole study cohort. Accordingly, apart from the group, significant positive correlations were found between intermediate CD14++CD16+ monocytes and the inflammatory markers ESR (ρ = 0.294, *p* = 0.02), CRP (ρ = 0.331, *p* = 0.008), and ferritin (ρ = 0.293, *p* = 0.02). The Treg percentage correlated negatively with serum triglyceride levels (ρ = −0.399, *p* = 0.001). The total lymphocyte counts correlated with serum albumin (r = 0.389, *p* = 0.04) and eGFR (r = 0.002, *p* = 0.04). Finally, NK cell number correlated positively with serum albumin (ρ = 0.388, *p* = 0.002). Notably, age and UPCR, parameters that differ significantly between the two groups, did not correlate with immune cell subsets in the whole study cohort or in each group separately. Following univariate regression analysis, the differences in immune cell subsets between the two groups remained statistically significant for CD14++CD16+ monocytes, total lymphocytes, and NK cells (*p* < 0.05 for all) but not for Tregs, after adjustment for various confounders including age. 

### 3.2. Immune Cell Subset Expression in Patients with Type 2 CRS 

Specifically, in patients with CRS-2, distinct monocyte subpopulations were found to be associated with inflammatory markers. Thus, we found a positive correlation between monocyte number with ESR (ρ = 0.485, *p* = 0.00) and CRP (ρ = 0.402, *p* = 0.001). Likewise, both the number and percentage of intermediate CD14++CD16+ monocytes correlated positively with CRP (ρ = 0.476, *p* = 0.002) and (ρ = 0.319, *p* = 0.040), respectively. Additionally, the number of the classical CD14++CD16- monocytes, as well as the intermediate CD14++CD16+ monocytes, correlated with ESR [(r = 0.353, *p* = 0.030 and (r = 0.378, *p* = 0.020), respectively]. On the other hand, serum hemoglobin levels displayed a negative association with both classical CD14++CD16- and nonclassical CD14+CD16++ monocyte counts (r = −0.332, *p* = 0.004) and (ρ = −0.385, *p* = 0.010), respectively. Regarding indices of kidney function, a positive correlation was found between eGFR and total lymphocytes (r = 0.427, *p* = 0.007), T-cells (r = 0.425, *p* = 0.007), as well as CD4+ T-cell counts (r = 0.439, *p* = 0.005), whereas the CD4+ T-cells to CD8+ T-cells ratio displayed a negative correlation with UPCR (ρ = 0.401, *p* = 0.02). Significant associations were detected between serum levels of total cholesterol and LDL cholesterol with B-lymphocyte counts (ρ = −0.336, *p* = 0.03) and (ρ = −0.388, *p* = 0.01), respectively and percentage of B-lymphocytes (ρ = −0.470, *p* = 0.003) and (ρ = −0.441, *p* = 0.0005), respectively. Additionally, both CD8+ T-cell number and percentage correlated positively with HDL cholesterol (ρ = 0.318, *p* = 0.04) and (ρ = 0.333, *p* = 0.04), respectively. Finally, a negative association was detected between Tregs and serum triglycerides in the whole study cohort, which was confirmed within the CRS-2 patient group as well (ρ = −0.377, *p* = 0.03) (Table 3). 

Regarding cardiac indices, a positive association was found between BNP levels and CD14++CD16- monocytes (r = 0.565, *p* = 0.02), whereas hsTnI levels correlated negatively with the percentage of total lymphocytes (r = −0.575, *p* = 0.006).

We further determined differences regarding the expression of immune cell subpopulations with respect to the clinical characteristics of CRS-2 patients. Accordingly, the number and percentage of nonclassical CD14+CD16++ monocytes were higher in CRS-2 patients with left ventricular EF less than 30% compared to patients with left ventricular EF above 30% [33 (IQR, 18–37)/µL versus 13 (IQR, 10–29)/µL (*p* = 0.02) and 4.5% (IQR, 3.4–7.2%) versus 2.7% (IQR, 1.9–5.4%) (*p* = 0.03), respectively]. With regard to CRS-2 etiological background, patients with nonatherosclerotic compared to patients with atherosclerotic CVD displayed increased counts of intermediate CD14++CD16+ monocytes [75 (IQR, 41–104)/µL versus 36 (IQR, 22–61)/µL (*p* = 0.01)] and nonclassical CD14+CD16++ monocytes [37 (IQR, 35–49)/µL versus 21 (IQR, 12–32)/µL (*p* = 0.02)]. After univariate regression analysis, the correlation between CD14++CD16+ monocytes and atherosclerotic CVD, as well as ESR, remained statistically significant (*p* = 0.01 and 0.030, respectively). Finally, NK cell and Treg levels were lower in patients with atrial fibrillation compared to those without [133 (IQR, 79–173)/µL versus 260 (IQR, 151–314)/µL (*p* = 0.01)] and [32 (IQR, 21–43)/µL vs. 47 (IQR, 34–85)/µL (*p* = 0.006)], respectively.

### 3.3. Survival Analyses in Type 2 CRS Patients Categorized by Circulating Immune Cell Subset Expression

During a median follow-up of 29.8 ± 3.4 months, 23 CRS-2 patients (59%) reached the study endpoint with no patients being lost to follow-up. At binary logistic regression analysis, immune cell subpopulations that correlated with all-cause and cardiovascular death included total lymphocyte counts (OR 0.85 per 100 cells/μL increase; 95% CI 0.75–0.97; *p* = 0.01), T-cell number (OR 0.82 per 100 cells/μL increase; 95% CI 0.70–0.96; *p* = 0.01), CD4+ T-lymphocyte number (OR 0.66 per 100 cells/μL increase; 95% CI 0.50–0.87; *p* = 0.004), CD8+ T-lymphocyte counts below their median value cut-off of 410/μL (OR 4.67; 95% CI 1.14–19.07; *p* = 0.03), Treg counts below their median value cut-off of 35/μL (OR 6.63; 95% CI 1.36–23.27; *p* = 0.01), and CD14++CD16+ monocyte counts above their median cut-off value of 40/μL (OR 4.13; 95% CI 1.06–16.1; *p* = 0.04). In a multivariate model including all six immune cell subsets, only the CD4+ T-lymphocytes remained independent predictors of mortality (OR 0.66; 95% CI 0.50–0.87; *p* = 0.004). In contrast, no such associations were found for age, eGFR, UPCR, hsTnI, BNP, as well as the rest of the clinical or laboratory indices.

**Figure 3:**
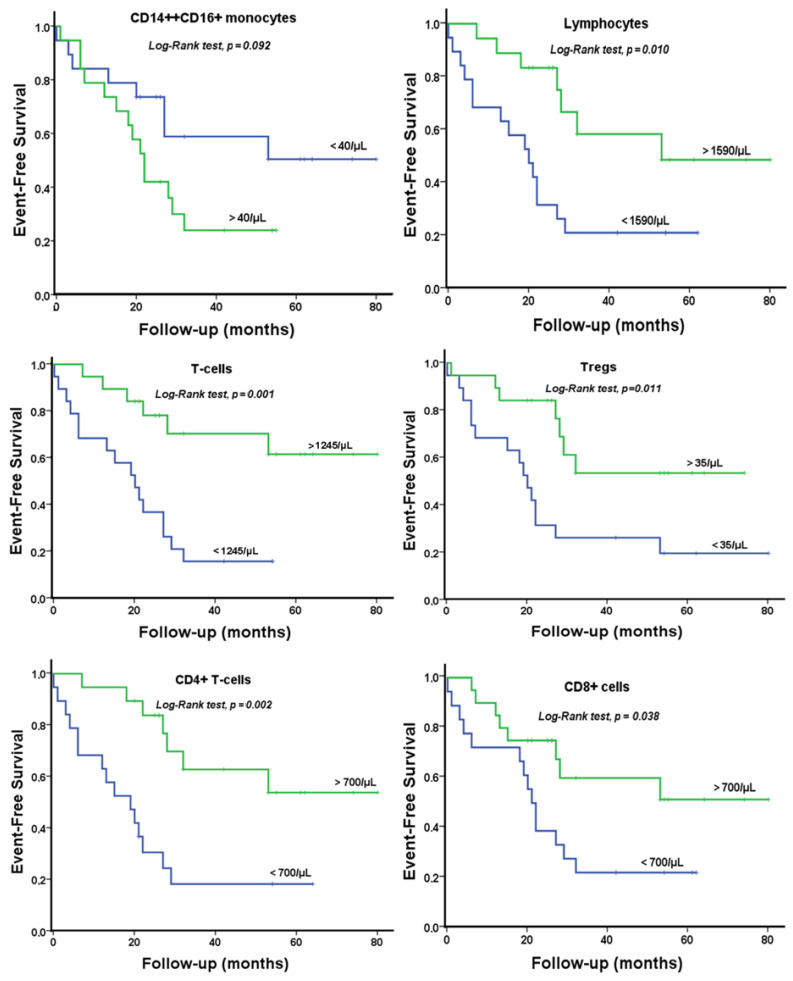
Kaplan–Meier curves of endpoint-free patients with circulating immune cell subset expression below or above median value derived cut-offs.

Subsequently, Kaplan–Meier survival curves for CRS-2 patients according to the levels of immune cell subpopulations (i.e., below vs. above median value) are shown in Figure 3. Decreased levels of lymphocytes, T-lymphocytes, CD4+ T-cells, CD8+ T-cells, and Tregs were associated with mortality at a median follow-up of 30 months (*p* < 0.05 for all log-rank tests). Increased levels of proinflammatory intermediate CD14++CD16+ monocyte counts showed a trend for increased mortality (*p* = 0.093). 

## 4. Discussion

To the best of our knowledge, this is the first report in the literature assessing and comparing the profile of immune cell subtypes, including the CD14++CD16+ proinflammatory monocyte subpopulation, NK cells, and lymphocyte subpopulations between patients with CRS-2 and patients with CKD but without established CVD. In addition, the potential associations of immune cells with clinical parameters, biomarkers, and outcomes have not been studied in this specific patient population until now.

Interestingly, we found increased levels of proinflammatory intermediate CD14++CD16+ monocytes in CRS-2 patients as compared to CKD patients, albeit no significant differences were detected between other robust markers of inflammation, such as CRP or ESR between the two cohorts. Although monocytes are considered an essential component of the inflammatory cascade in heart failure, relevant evidence until now has been based mainly on studies regarding monocyte-derived cytokines implicated in the pathogenesis of heart failures, such as TNF and IL-6 [26,27]. There are few and relatively recently published studies that have found elevated CD14++CD16+ monocyte counts in patients with both acute and stable chronic heart failure as well as an association of these proinflammatory monocytes with heart failure severity and decreased GFR levels [28,29,30]. Likewise, in CKD patients, the intermediate monocyte counts are higher than those in healthy subjects and appear to decrease early after transplantation [31,32,33]. Thus, according to our findings, subtle and possibly additive immune mechanisms might further aggravate the proinflammatory milieu of CKD in the setting of heart failure.

Moreover, our results indicate a potential association between the proinflammatory CD14++CD16 monocytes and adverse outcomes in CRS-2 patients. This is in accordance with evidence from previous studies revealing that higher counts of CD14++CD16+ monocytes are associated with incident cardiovascular events and death in high atherosclerotic risk populations, including non-end-stage CKD and hemodialysis patients [31,34,35]. With regard to the etiology of heart failure, we found increased levels of CD16-positive monocytes, that is, both CD14++CD16+ and CD14+CD16++ monocytes in patients with nonatherosclerotic CVD. However, attempts to justify this finding would be speculative at present, considering the paucity of relevant data in the literature. Thus, until very recently, the evidence for the pathophysiological implication of monocytes in heart failure stems from heart failure with reduced EF of mixed etiology [28,36]. Accordingly, patients with ischemic heart failure display similar levels of classical monocytes and increased levels of intermediate monocytes compared to patients with coronary artery disease and without heart failure, whereas the status of nonclassical monocytes remains controversial [28,36,37]. Regarding the last, we found an inverse association between nonclassical monocytes and left ventricular EF in CRS-2 patients. Finally, recent findings suggest that raised intermediate monocyte counts are observed in heart failure with preserved EF as well [38].

Our results showed lower levels of NK cells in patients with CRS-2 compared to their CKD counterparts as well as in CRS-2 patients with chronic atrial fibrillation compared to those without. The depleted NK cell levels in heart failure and advanced CKD have been ascribed to the chronic inflammatory state and upregulation of IL-6 pathways which induces NK cell dysfunction and anergy [39]. Experimental models of viral myocarditis have shown that NK cells might have a protective role against cardiac fibrosis by limiting eosinophilic infiltration and affecting chemokine production by cardiac fibroblasts, whereas their beneficial versus injurious role in atherosclerotic plaques is a subject of dispute [19,40]. However, a recent analysis of the relationship between immune cell subsets and hypertension from the multi-ethnic study of atherosclerosis (MESA) showed that increments in NK cell levels were associated with a higher average level of systolic blood pressure. Furthermore, it should be noted that studies investigating NK cells in CKD patients are few, conducted in small cohorts, and have mainly analyzed NK cells in hemodialysis patients. Thus, in a previous study conducted by our group in a cohort of peritoneal dialysis patients, increased NK cells were linked to fluid overload determined either as overhydration in lung ultrasound, body composition monitor measurements, or an increased E/E’ in echocardiography [32]. As a result, further research is to clarify and specify the role of NK cells within the heterogenous pathological entities included in the CVD and CKD spectrum.

The results of our study showed lower levels of lymphocytes, as well as an inverse association of lymphocyte counts with mortality in CRS-2 patients. Likewise, levels of lymphocyte subpopulations and specifically T-lymphocytes, CD4+ T-cells, and CD8+ T-cells, were associated with survival in our cohort of CRS-2 patients. Our results pair well with those from previous studies linking reduced lymphocyte count with adverse prognosis in heart failure patients [41]. Notably, in the multivariate model including all immune cell subtypes associated with the prospective endpoint of our study, only lower levels of CD4+ T-lymphocytes remained independent predictors of mortality. With regard to indices of kidney function, we showed that eGFR levels in CRS-2 patients were positively associated with total lymphocytes, T-lymphocytes as well as CD4+ T-cell counts, which are in line with available findings from studies conducted in patients with advanced stages of CKD [15]. Furthermore, we detected an inverse association between the ratios of CD4+ T-cells to CD8+ T-cells with proteinuria in CRS-2 patients, despite overall low mean levels of proteinuria in patients with CRS-2 compared to CKD patients. Considering that proteinuria is a marker for both progression of CKD and increased cardiovascular morbidity and mortality, larger studies are needed in the future to clarify the pathophysiological and prognostic role of these findings [42]. 

Interestingly, we found that lower B-lymphocyte counts were associated with an adverse lipid profile. Recent data indicate that B-cells regulate atherosclerotic plaque formation through the production of antibodies and cytokines, and their effects are subset-specific; thus, future research will further evaluate their role in atherogenesis [43]. 

The role of Tregs in CVD and CKD has attracted a great deal of research interest during the last few years; however, the available evidence is controversial. We found decreased levels of Tregs in patients with CRS-2, although the association was lost after correcting triglyceride levels. Moreover, lower levels of Tregs were observed in patients with CRS-2 and atrial fibrillation. Initial studies showed a depletion of Tregs in patients with heart failure with reduced EF. However, recent data indicate that there is a change in the Treg phenotype towards a profibrotic one in chronic heart failure [44,45,46]. 

Our study has strengths and limitations, which should be noted. The main strength of our study is its design, including a homogeneous cohort of patients with CRS-2 in terms of clinical characteristics and a gender and eGFR-matched CKD cohort for making adequate comparisons. In addition, the prospective arm of the study with an appropriate follow-up duration and an inclusive combined endpoint allowed us to examine and determine the predictive value of immune cell subsets for hard adverse outcomes. The key limitations of our study are the small sample size, gender limitations, and its observational nature, which does not allow us to confirm causality in the associations found between circulating immune cells, clinical variables, and patient outcomes. 

## 5. Conclusions

Patients with CRS-2 exhibit alterations of the immune cell subsets profile in the circulation compared to CKD patients of similar kidney function but without established cardiovascular disease. Our findings suggest that distinct immune mechanisms might be involved in the pathogenesis or during the chronic clinical course of CKD in the setting of heart failure as compared to CKD without established CVD. Future research is required to elucidate further and specify the pathophysiological role of immune cell subpopulations as well as evaluate their potential value as markers of prognostic significance.

## Figures and Tables

**Table 1:** Main clinical characteristics in all patients, in patients with type 2 CRS, and in CKD patients. Statistically significant differences between subgroups are highlighted in bold.

	All Patients (N = 63)	CRS Patients (N = 39)	CKD Patients (N = 24)	*p*-Value *
Age (years)		72 ± 10	66 ± 10	**0.01**
DM, N (%)	31 (47.7)	22 (56.4)	9 (37.5)	0.15
eGFR (mL/min/1.73 m^2^)	35 ± 15	37 ± 14	33 ± 16	0.18
UPCR (g protein/g creatinine)	0.39 (0.12–1.13)	0.19 (0.10–0.52)	1.03 (0.17–2.09)	**0.02**
Hemoglobin (g/dL)	12.3(11.0–14.4)	11.8 (11.0–14.4)	13 (11.1–14.6)	0.78
Uric Acid (mg/dL)	7.0 (5.9–8.4)	6.9 (5.5–7.8)	7.9 (6.05–8.9)	0.17
ESR (mm/h)	33 ± 18	33 ± 19	32 ± 15	0.87
CRP (mg/L)	4 (2–8)	4 (2–8)	4 (3–8)	0.92
Glucose (mg/dL)	107 (93–137)	120 (98–160)	99 (91–113)	**0.007**
Albumin (g/dL)	4 (3.7–4.4)	3.9 (3.7–4.3)	4.2 (3.7–4.5)	0.26
Total Proteins (g/dL)	7.0 (6.4–7.6)	6.9 (6.3–7.5)	7.2 (6.7–7.6)	0.17
Total cholesterol (mg/dL)	161 ± 44	147 ± 40	184 ± 41	**0.001**
Triglycerides (mg/dL)	125 (94–178)	104 (77–155)	150 (118–216)	**0.006**
LDL cholesterol (mg/dL)	94 ± 40	84 ± 35	110 ± 44	**0.01**
HDL cholesterol (mg/dL)	38 ± 10	37 ± 11	40 ± 10	0.29
Ferritin (ng/mL)	76 (43–114)	63 (36–115)	88 (58–112)	0.31
Calcium (mg/dL)	9.4 (9.0–9.7)	9.4 (9.1–9.7)	9.4 (8.7–9.6)	0.16
Phosphorus (mg/dL)	3.7 ± 0.7	3.7 ± 0.6	3.7 ± 0.8	0.83
iPTH (pg/mL)	130 (86–235)	134 (92–176)	128 (62–281)	0.55
hsTNI (ng/mL)	/	25.3 (16.4–42.4)	/	/
BNP (pg/mL)	/	324 (184–797)	/	/
Statins N (%)	49 (79.0)	32 (84.2)	17 (70.8)	0.22
ACEI/ARB N (%)	30 (48.4)	14 (36.8)	16 (66.7)	**0.02**
B-blockers N (%)	44 (71)	34 (89.5)	10 (41.7)	**0.000**

Values are expressed in mean (± SD) or median (IQR 25–75th percentiles). ACEI/ARB, angiotensin-converting enzyme inhibitors/angiotensin receptor blockers; CKD, chronic kidney disease; CRP, C-reactive protein; CRS, cardiorenal syndrome; eGFR, estimated glomerular filtration rate; ESR, erythrocyte sedimentation rate; hs-TNI, high sensitivity troponin I; iPTH, intact parathyroid hormone; N, number; NT-proBNP, N-terminal prohormone BNP; UPCR, urinary protein to creatinine ratio. * *p* refers to *t*-test significance for normal distribution variables, Mann–Whitney test significance for nonparametric variables, or to chi-square test significance for categorical variables.

**Table 2:** Immune cell subpopulations in all patients, patients with type 2 CRS, and CKD patients. Statistically significant differences between subgroups are highlighted in bold.

	All Patients (N = 63)	CRS Patients (N = 39)	CKD Patients (N = 24)	*p*-Value *
WBC (N)	7730 (IQR 6224–9495)	8360 (IQR 6730–9940)	7330 (IQR 6070–8830)	0.15
Monocytes (N)	500 (IQR 400–650)	600 (IQR 400–700)	500 (IQR 400–600)	0.06
Monocytes (%)	6.5 (5.4–7.9)	6.5 (IQR 5.4–8.1)	6.6 (IQR 5.3–7.8)	0.64
CD14++CD16- (N)	427 ± 167	450 ± 184	391 ± 132	0.14
CD14++CD16- (%)	81.4 ± 8.9	80.6 ± 10	82.6 ± 6.9	0.35
CD14++CD16+ (N)	38 (IQR 22–62)	41 (IQR 24–78)	35 (IQR 18–43)	**0.04**
CD14++CD16+ (%)	7.4 (IQR 5.4–11.2)	8 (IQR 5.6–12.0)	7.3 (IQR 4.7–9.6)	0.30
CD14+CD16++ (N)	25 (IQR 14–35)	22 (IQR 12–36)	25 (IQR 19–32)	0.80
CD14+CD16++ (%)	4.6 (IQR 3.0–6.7)	4.2 (IQR 2.7–6.6)	5.1 (IQR 4.0–6.7)	0.14
Lymphocytes (N)	1699 ± 658	1557 ± 691	1920 ± 545	**0.03**
Lymphocytes (%)	21.3 ± 8.7	18.7 ± 8.3	25.3 ± 8.0	**0.002**
T-lymphocytes (N)	1320 ± 500	1227 ± 510	1465 ± 455	0.06
T-lymphocytes (%)	79.6 ± 9.7	81.7 ± 8.7	76.3 ± 10.3	**0.03**
B-lymphocytes (N)	75 (IQR 37–140)	68 (IQR 31–104)	87 (IQR 58–163)	0.08
B-lymphocytes (%)	4.7 (2.9–8.3)	4.2 (IQR 2.2–9.0)	5.1 (IQR 3.4–7.9)	0.57
NK cells (N)	182 (124–328)	148 (IQR 103–258)	324 (IQR 179–368)	**0.001**
NK cells (%)	1.7 (IQR 8.2–18.3)	10.7 (IQR 7.1–16.6)	16.5 (IQR 11.2–19.6)	**0.01**
CD4+ T-Cells (N)	787 ± 312	732 ± 308	873 ± 304	0.08
CD4+ T-cells (%)	47.5 ± 10.6	48.6 ± 10.4	45.7 ± 10.9	0.30
CD8+ T-cells (N)	508 (IQR 353–750)	411 (IQR 224–720)	585 (IQR 447–786)	0.14
CD8+ T-cells (%)	29.9 (IQR 23.5–37.9)	28.5 (IQR 23.3–38.0)	31.5 (IQR 24.4–36.8)	0.73
Tregs (%)	2.4 (IQR 1.7–3.3)	2.7 (IQR 2.0–3.9)	2.0 (IQR 1.6–2.6)	**0.03**
T Regs (N)	37 (IQR 25–51)	36 (IQR 24–49)	40 (IQR 26–61)	0.94

Values are expressed in mean (± SD) or median (IQR 25–75th percentiles). CD, cluster of differentiation; NK, natural killer; N, number; Tregs, T regulatory cells. * *p* values refer to *t*-test significance for normal distribution variables, Mann–Whitney test significance for nonparametric variables, or chi-square test significance for categorical variables.

**Table 3:** Associations of immune cell subpopulations in patients with type 2 CRS with inflammatory markers, eGFR, and serum lipid levels.

**Monocytes (N)**	**Lymphocytes (N)**	**CD8+ T-cells (%)**
ESR r = 0.485 *p* = 0.00	eGFR r = 0.427 *p =* 0.007	HDL cholesterol r = 0.333, *p =* 0.04
CRP r = 0.402 *p =* 0.001		
**CD14++CD16- (N)**	**T-lymphocytes (N)**	**B-lymphocytes (N)**
ESR r = 0.353, *p =* 0.030	eGFR r = 0.425 *p =* 0.007	Total cholesterol r = −0.336 *p =* 0.03
		LDL cholesterol r = −0.388, *p* = 0.01
**CD14++CD16+ (N)**	**CD4+ T-cells (N)**	**B-lymphocytes **(%)
ESR r = 0.378, *p =* 0.020	eGFR r = 0.439 *p =* 0.005	Total cholesterol r = −0.470 *p =* 0.003
CRP r = 0.476 *p =* 0.002		LDL cholesterol r = −0.441, *p* = 0.0005
**CD14++CD16+ (%)**	**CD8+ T-cells (N)**	**Tregs (%)**
CRP r = 0.319, *p* = 0.040	HDL cholesterol r = 0.318, *p =* 0.04	Triglycerides r = −0.377, *p =* 0.03

Correlations were assessed by Spearman’s or Pearson’s rank tests. Only correlations reaching statistical significance are presented. CRP, C-reactive protein; CRS, cardiorenal syndrome; eGFR, estimated glomerular filtration rate; ESR, erythrocyte sedimentation rate.

## Data Availability

Main relevant data is contained within the article. Additional data regarding methods and results presented in this study are available on request from the corresponding author.
